# Common childhood kidney diseases in Uganda and their prevention

**DOI:** 10.4314/ahs.v17i4.21

**Published:** 2017-12

**Authors:** Amos Odiit

**Affiliations:** Mulago Hospital, Paediatrics & Child Health; Makerere University, College of Health Sciences, Paediatrics & Child Health

Dear Editor,

The 10^th^ March 2016 was World Kidney day, with the theme:'Kidney disease and children. Act early to prevent it'^1^. In Kampala, the capital city of Uganda, the event was hosted by Makerere University College of Health Sciences (MUCHS), and was graced by the representative of the University's Vice Chancellor. That this was the first ever World Kidney day theme to focus on Paediatric kidney disease was remarked by one of the presenters. The presentations included: Paediatric and adult nephrology, Paediatric Urology, and one from the Uganda Kidney Psychosocial Support Organization. The meeting keenly listened to a mother of a paediatric patient with steroid resistant nephrotic syndrome who had been under care for the past 8 years.

The mother narrated her interface with the health service in the care of her daughter whose histological diagnosis was membranous nephropathy. The child was notable for short stature compared to her twin sibling. Another presentation was from the spouse of a kidney transplant patient who shared her family's struggles before and after transplantation. Medical students of MUCHS set up tents for screening for kidney disease. They examined urine for protein, measured body mass index, and blood pressure.

It is my intention in this letter to reflect on some of the common paediatric kidney diseases in Uganda and the region, underlining the potential for prevention.

Starting at the perinatal period, congenital anomalies of the Kidney and Urinary Tract (CAKUT) are a group of conditions that cause significant morbidity and mortality in children^2^. Though facilities for detailed genetic studies are usually not available for day to day use in clinical practice, pre-marital screening for CAKUT by taking careful family history should be possible. Through family history and pre-marital counseling, primary prevention should be possible for polycystic kidney disease, and Alport's syndrome. Antenatal ultrasound and physical examination should enable early diagnosis of pelvi-ureteric junction obstruction, and posterior urethral valves. Early surgical intervention for the identified cases prevents progress of kidney pathology, and constitutes secondary prevention of AKI and Chronic Kidney Disease (CKD).

Acute Kidney Injury (AKI) occurring due to birth asphyxia is another important condition early in life. In Nairobi, Kenya, 36.6% of the children with birth asphyxia developed AKI^3^. In the Democratic Republic of the Congo 42.9% of sick neonates had AKI^4^. The magnitude of AKI due to birth asphyxia is most likely similar in other resource-constrained countries. In contrast to the high prevalence of AKI in sub-Saharan countries, AKI was present in only 2.7% of neonates in an Iranian tertiary hospital^5^. Yet birth asphyxia, and therefore AKI is preventable through good antenatal care and delivery (primary prevention). Babies who have been through difficult delivery should be watched for AKI through monitoring of urinary output, and by measuring serum creatinine. Once perinatal AKI is detected early, secondary prevention should include avoidance of nephrotoxic drugs, for instance gentamycin, which is often used for presumed neonatal sepsis. Other secondary preventive measures for neonatal AKI are: giving appropriate amounts of fluids, and doing dialysis when necessary.

Another sub-grouping of kidney diseases afflicting children in Uganda is acute kidney injury following acute severe infections. Of 2055 children presenting to hospital in Uganda with either: severe acute gastroenteritis, severe pneumonia or severe malaria, 13.5% had AKI^6^. Each of these 3 conditions associated with AKI is preventable through various ways. Primary prevention of AKI in this respect includes: provision of safe and adequate water to households, health education on personal and food hygiene, childhood vaccination, use of oral rehydration salts, and use of insecticide treated nets.

When AKI has occurred, prompt and adequate hydration may improve kidney function. Yet very often in Uganda, a child with gastroenteritis reaches a health facility without having taken any appropriate fluids. This reflects lack of knowledge of how to manage acute diarrhoea at community level. Malaria control is feasible through use of insecticide treated nets (ITN's), and malaria case management, in addition to other interventions. Despite the demonstrated usefulness of ITN's in malaria control, their coverage in sub-Saharan Africa varies widely, from 3% to 80%^7^. Cultural practices and community activities may encourage spread of diseases that are associated with AKI.

Some cultural festivities involve gathering of large numbers of people under inadequate sanitary conditions. When the festivities happen through the night, the celebrants, including children are exposed to mosquitoes. Sustained sensitization of the masses on malaria prevention should be possible as a primary preventive measure for AKI. Pneumonia, on the other hand is preventable, at least partly through *Streptococcus pneumoniae* and *Haemophilus* type B vaccination. Ensuring adequate child nutrition through health education and dietary treatment of malnutrition will contribute to primary prevention of AKI since hypoproteinaemia is associated with AKI^6^.

Nephrotic syndrome: In Uganda, the incidence of proteinuric kidney disease, in children is estimated at 160/million population, which is at least 8 times the incidence in the United Kingdom^8^. Nephrotic syndrome may be idiopathic, or secondary to specific disease entities such as *Plasmodium malariae*^9^, Hepatitis B, HIV infection, Hodgkin's lymphoma, and Syphilis. The secondary causes of nephrotic syndrome abound in sub-Saharan Africa, and most of them are either treatable or preventable.

Acute Post-*Streptococcal* Glomerulonephritis (APSGN) has an incidence varying from 9.5 to 28/100,000 population/year^10^. It has virtually disappeared from developed countries following the industrial revolution. The picture of APSGN is different in developing countries. At the Red Cross Children's Hospital in South Africa, 110 cases of APSGN were diagnosed over a period of 20 months. More than 50% of the cases had a recent history of skin sepsis with or without scabies. Both scabies and bacterial skin infection are diseases predominantly of poor communities^11^. Improvement in people's living standards, as a primary preventive measure will reduce PSGN. When APSGN has already occurred, early identification followed by careful management of blood pressure, fluids, electrolytes, and nitrogen balance constitute secondary preventive measures against further kidney damage.

Acute Kidney Injury may follow massive release of haemoglobin into the circulation, which may occur in Glucose 6 Phosphate Dehydrogenase (G-6PD) deficiency and during the haemolytic crisis of Sickle cell anaemia (SCA)^12^. Avoidance (whenever possible) of quinine and sulphur drugs in children with G-6PD deficiency primarily prevents AKI from occurring. Early recognition of AKI, treatment of infections, and correction of anaemia are the secondary preventive measures for AKI in G-6PD deficiency. Among children with SCA, the prevalence of nephropathy in Ugandan children was found to be 28.2%^13^. Malaria prophylaxis, *pneumococcal* vaccine and other vaccinations, keeping normal hydration status, maintaining steady state haemoglobin, and treating acute infections are primarily preventive of AKI in SCA. When nephropathy has already set in, Angiotensin Converting Enzyme Inhibitors (ACEI) may be used to slow down progress of nephropathy.

A silent but important cause of Kidney disease in sub-Saharan Africa children is Human Immunodeficiency Virus (HIV) infection. It is estimated that 30% of HIV infected individuals end up with Kidney disease^14^. Use of Highly Active Anti-Retroviral Therapy (HAART) in HIV infected children has reduced mortality and primarily prevented development of HIV-associated kidney diseases, including AKI^15^. Enrolling HIV infected children to HIV care and careful dosing of anti-retroviral drugs, are primary preventive measures against AKI^16^, while regular monitoring of kidney functions will enable early detection of AKI and permit appropriate intervention. In Rwanda, 30.2% of HIV infected children, most of them on HAART, had proteinuria, evidence of CKD^17^. Such children who are proteinuric while on HAART require early detection and treatment with Angiotensin Converting Enzyme Inhibitors^14^, as a secondary preventive measure for kidney disease.

Hypertension, primary or secondary may occur in children. Up to 3.8% of Ugandan school going children were found with persistent hypertension^18^. Considering the whole country, 3.8% is a large number of children given that about 1/2 the Ugandan population is children. Primary prevention of kidney disease from hypertension involves early recognition and treatment of hypertension. In hypertensive children, regular health checks, including serum creatinine, are required for early detection of AKI, and institution of early appropriate treatments. Detection of childhood hypertension through school health programs, and during the medical examination of school children when returning to school should be possible. Yet parents, children and the health workers do not always take those opportunities seriously enough.

Children are increasingly being diagnosed with diabetes mellitus (DM).While nephropathy complicating DM usually starts about 5 years after onset of the disease^19^, AKI may occur at any time DM is out of control, usually due to severe dehydration. Strict maintenance of blood sugar levels is known to prevent or at least delay the onset of diabetic nephropathy^19^.

Urinary tract infections (UTI) occur frequently in children. At Mulago hospital, 13% of the children presenting with fever had UTI^20^. Sepsis from upper UTI may cause AKI. Repeated or untreated upper UTI may progress to chronic pyelonephritis and CKD. Early detection of UTI may be achieved by screening for UTI in all children with fever who have no apparent focus of infection. Furthermore, urinary tract infection is prevented by avoiding unnecessary urinary tract catheterization, observing infection control measures in hospital, vaccinating against the vaccine preventable causes of UTI, for example *Strep. pneumoniae*. By treating the various causes of immunosuppression in children, and surgically correcting urinary tract anomalies, such as vesico-ureteric reflux, and posterior urethral valves, UTI may be prevented.

Tertiary prevention of kidney disease aims at limiting the effects of ongoing kidney pathology. It is more specialized and expensive. It may involve: surgery to partially or totally relieve an obstruction in the urinary tract, use of immunosuppressive drugs in rapidly progressive glomerulonephritis, or renal replacement therapy, including transplantation. Elaborate laboratory and radiological services are vital for tertiary care of kidney disease.

In summary, many child-hood diseases in Uganda and sub-Saharan Africa may lead to kidney disease; but most of such diseases are either preventable or treatable, making kidney disease in children potentially preventable. On the other hand, tertiary prevention is more complex and less available in Uganda and the region.
